# Activation in inhibitory brain regions during food choice correlates with temptation strength and self-regulatory success in weight-concerned women

**DOI:** 10.3389/fnins.2014.00308

**Published:** 2014-09-30

**Authors:** Laura Nynke van der Laan, Denise T. D. de Ridder, Max A. Viergever, Paul A. M. Smeets

**Affiliations:** ^1^Image Sciences Institute, University Medical Center UtrechtUtrecht, Netherlands; ^2^Department of Clinical and Health Psychology, Utrecht UniversityUtrecht, Netherlands; ^3^Division of Human Nutrition, Wageningen University and Research CentreWageningen, Netherlands

**Keywords:** fMRI, food choice, self-regulation, dietary restraint, orbitofrontal cortex, supplementary motor area (SMA)

## Abstract

Food choices constitute a classic self-control dilemma involving the trade-off between immediate eating enjoyment and the long term goal of being slim and healthy, especially for weight-concerned women. For them, decision-making concerning high (HE) and low energy (LE) snacks differs when it comes to the need for self-control. In line, our first study aim was to investigate which brain regions are activated during food choices during HE compared to LE energy snacks in weight-concerned women. Since it is particularly difficult to resist HE snacks when they are very tasty, our second aim was to investigate in which brain regions choice-related activation varies with the food's tastiness. Our third aim was to assess in which brain regions choice-related activation varies with individual differences in self-regulatory success. To this end, 20 weight-concerned women indicated for 100 HE or LE snacks whether they wanted to eat them or not, while their brains were scanned using fMRI. HE snacks were refused more often than equally-liked LE snacks. HE snack choice elicited stronger activation in reward-related brain regions [medial to middle orbitofrontal cortex (OFC), caudate]. Highly tasty HE snacks were more difficult to resist and, accordingly, activation in inhibitory areas (inferior frontal gyrus, lateral OFC) was negatively associated with tastiness. More successful self-controllers showed increased activation in the supplementary motor area during HE food choices. In sum, the results suggest that HE snacks constitute a higher reward for weight-concerned women compared to (equally-liked) LE snacks, and that activation during food choice in brain regions involved in response inhibition varied with tastiness and individual differences in self-regulatory success. These findings advance our understanding of the neural correlates of food choice and point to new avenues for investigating explanations for self-regulatory failure.

## Introduction

Approximately 50% of the Western female population reports to be concerned with their weight, to be a regular dieter, or to attempt to limit food intake (Rideout and Barr, 2009; Fayet et al., 2012; de Ridder et al., 2014). However, in contrast to what their alleged weight concerns suggest, empirical evidence shows that self-reported weight-concerned women do not eat less than their non-weight-concerned counterparts and some studies even suggest that they are at increased risk for weight gain (French et al., 1994; Stice et al., 2004, 2007, 2010; Mann et al., 2007; de Witt Huberts et al., 2013).

For weight-concerned individuals, food choices constitute a classic self-control dilemma involving the trade-off between immediate eating enjoyment and the long term goal of being slim and healthy (Fishbach et al., 2003). Eating low-energy (LE) snacks is congruent with that goal. Since eating high-energy (HE) snacks is not, these individuals should exercise self-control to resist the HE snack in order to adhere to their long term goal. The need for self-control is particularly strong when HE snacks are very tasty. From the latter it follows that for weight-concerned women decision-making concerning HE and LE snacks differs when it comes to the need for self-control. Food-related decisions are made in the brain. For health promotion purposes it is therefore vital to increase understanding of the neural correlates of food choice in weight-concerned women. Insight into differential neural responses during food choices concerning HE and LE foods might explain why it is so difficult for weight-concerned women to adhere to their long-term goal.

To date, the neural responses during food choice in weight-concerned women have received relatively little attention. In contrast, the brain responses during viewing (HE and LE) foods and during food choice have been studied extensively in the general population and in selected non-weight-concerned populations (e.g., St-Onge et al., 2005; Uher et al., 2006; Frank et al., 2010; Hare et al., 2011; van der Laan et al., 2011, 2012; Smeets et al., 2013). However, studies in non-weight-concerned individuals do not provide insights into self-control because for them energy content does not play a prominent role in food choice (Arvola et al., 1999; Ayres et al., 2012). A HE snack will only trigger the need for self-control if someone actually has the long-term goal to restrict intake (Fishbach et al., 2003).

Only a few studies have assessed the neural responses to foods in weight-concerned individuals (Coletta et al., 2009; Hare et al., 2009; Born et al., 2011; Burger and Stice, 2011; Demos et al., 2011; Wagner et al., 2012; van der Laan et al., 2014). Most of these studies assessed the neural responses during passive viewing rather than during food choice: these studies showed that weight-concerned individuals have stronger activation in areas involved in food reward [e.g., striatum, orbitofrontal cortex (OFC)] and response inhibition (inferior frontal gyrus) when viewing food (compared to nonfood) (Coletta et al., 2009; Demos et al., 2011; Wagner et al., 2012), although null-findings have also appeared (Burger and Stice, 2011). To our knowledge, none of these studies assessed the contrast of viewing HE vs. LE foods. Thus, it is unknown in how far weight-concerned women respond differently to HE and LE foods. Two earlier studies have investigated the neural correlates of food choice in weight-concerned women (Hare et al., 2009; van der Laan et al., 2014). Hare et al. (2009) found that both the healthiness (strongly related to energy content) and tastiness ratings of foods correlated with activation in the vmPFC during food choice and they suggest that for a self-control attempt to be successful there should be dlPFC activation at the moment of choice to incorporate healthiness considerations. We previously found that weight-concerned women are generally unsuccessful in choosing LE over HE snacks and that this might be explained by a lack of anterior cingulate activation in response to the self-control dilemma (van der Laan et al., 2014).

In these two earlier studies on food-related self-control in weight-concerned women, the choices were always between two foods (Hare et al., 2009; van der Laan et al., 2014). The evaluation of alternatives in binary or multiple choices differs from single choices in several aspects. Firstly, in multiple choices the calculated value of alternatives is always relative to the other options while in single food choices the alternative is evaluated on its own (De Martino et al., 2009; van der Laan et al., 2012). Secondly, the presence of other (e.g., healthy) alternatives can influence the perception of the self-control dilemma: vicarious goal fulfillment theory posits that the mere presence of a healthy option can fulfill health-related goals (Wilcox et al., 2009), irrespective of whether the healthy option is actually chosen or not, and thereby increase the chance of indulging in HE snacks (Chandon and Wansink, 2007; Fishbach and Zhang, 2008; Wilcox et al., 2009). To our knowledge, no earlier study investigated the neural correlates of food-related self-control in weight-concerned women in a single-choice paradigm, i.e., in the absence of other alternatives that might influence choices and accompanying neural responses.

Therefore, the present study assessed the neural correlates of single food choices in weight-concerned women. As outlined above, for these women decision-making differs between HE and LE snacks when it comes to the need for self-control. Accordingly, the first aim of this study was to investigate which brain regions involved in self-control are activated during decision-making concerning single HE compared to LE snacks, in weight-concerned women. Because refusing HE snacks is increasingly difficult with increasing tastiness, we expected that activation during HE (but not LE) snack choice in brain regions involved in conflict and self-control would vary with the snacks' tastiness. Therefore, our second aim was to establish how tastiness varies with brain activation during food choice for HE and LE snacks.

Weight-concerned women are a heterogeneous group varying greatly in self-regulatory success (Jansen et al., 2009; Keller and Siegrist, 2014). Earlier studies have shown that successful restraint eaters and successful dieters differ from unsuccessful counterparts in several aspects: they are better at inhibitory control tasks, they score higher on dispositional self-control and they have stronger automatic activation of long term goal activation when confronted with temptation (Papies et al., 2008; Kroese et al., 2011; Hofmann et al., 2014; Keller and Siegrist, 2014). Therefore, our third aim was to investigate in which brain regions activation during food choice covaries with individual differences in self-regulatory success (indicated by the amount of refused HE snacks).

## Materials and methods

### Ethics statement

The study was approved by the Medical Ethical Committee of the University Medical Center Utrecht (file 10-461) and subjects provided written informed consent.

### Participants

The study comprised of 20 women as participants (age in years: *M* = 21.2, *SD* = 2.8; BMI in kg/m^2^: *M* = 21.3, *SD* = 1.7). Participants filled in a questionnaire on in-/exclusion criteria upon screening. As self-control conflict is only relevant for individuals who are weight-concerned, inclusion criteria consisted of a restraint-score above average or high [Dutch Eating behavior questionnaire reference table for female students; Van Strien et al., 1986, (*M* = 3.1, *SD* = 0.4)] and a rating of 6 or higher on each of two questions: “To what extent are you weight-concerned?” (*M* = 6.6, *SD* = 0.8) and “To what extent are you occupied with being slim?” (*M* = 7.0, *SD* = 0.8; ranging from 1 = not at all to 9 = very much; adapted from Fishbach et al., 2003). Participant selection was limited to women because they generally score higher on weight concern and because of known gender differences in reasons for dieting as well as in brain anatomy and function (Pingitore et al., 1997; Neumark-Sztainer et al., 1999; Cahill, 2006; Luders et al., 2009). In addition, there are gender differences in the brain response to food cues (Smeets et al., 2006; Cornier et al., 2010; Frank et al., 2010; Haase et al., 2011). Additional inclusion criteria were having an age between 18 and 30 years, being right-handed and having a normal weight (BMI between 18.5 and 25 kg/m^2^). We included women with a healthy weight because research showed that normal-weight individuals who report high weight-concerns might be at increased risk for gaining weight (French et al., 1994; Mann et al., 2007). Exclusion criteria consisted of having a food allergy, having an eating disorder [“Do you have an eating disorder (for example, anorexia or bulimia nervosa)? Yes/No”], and having a history of medical or surgical events that might significantly affect the study outcome, such as metabolic or endocrine disease, or any gastro-intestinal disorder. Smokers and individuals having a current alcohol consumption of >28 units per week were excluded because these factors have been shown to affect the neural response to rewarding stimuli: heavy drinkers have reduced responses to food cues (Ihssen et al., 2011). Twenty eight units used to be the cut-off for “sensible” alcohol use, as defined by the British Royal College of Physicians. We excluded women that followed a medically prescribed diet in the past 6 months or that had weight fluctuations of more than 5 kg in the past 6 months so as to exclude participants who may show biases in their food choices for medical reasons. Participants were recruited with posters at the University Medical Center Utrecht, The Netherlands and the adjacent university campus.

### Study procedures

The study consisted of two sessions. During the first session, participants evaluated the expected tastiness and perceived energy of all food stimuli (presented on pictures) on 9-point scales ranging from 1 = very untasty / very few calories to 9 = very tasty / very many calories. To make sure participants were craving for a snack, they were instructed to refrain from eating and drinking (except water) for at least 2 h prior to both sessions but to have preferably eaten a meal within 2–3 h before the session (second session: mean time since last food intake in minutes ± *SD*: 140 ± 22). Moreover, to avoid effects of time of day, we planned the first and second session at approximately the same time of day. Upon arrival at the second session, participants received instructions and rated hunger on a VAS scale ranging from 0 (not hungry) to 100 (very hungry) (mean hunger ± *SD*: 59 ± 12). To ensure the relevance of their weight-concerns, participants filled out a questionnaire about an allegedly new type of biscuit (giving ratings of expected tastiness, expected energy content, and to what extent eating the biscuit is appropriate for individuals who are watching their weight). Next, participants were scanned using functional Magnetic Resonance Imaging (fMRI) while performing a food choice task. After the food choice task reported here, they performed another unrelated food choice task. At the end of the second session, participants received a snack of their choice, were thanked, and reimbursed.

### Stimuli

The visual stimuli consisted of 100 pictures of regularly available snack foods on plates with a gray background: 50 HE foods (energy content in kcal/100 gram: *M* = 419, *SD* = 103) and 50 LE foods (*M* = 56, *SD* = 37). The mean perceived energy content (rated in the first session on a 9-point scale ranging from 1 = very few calories to 9 = very many calories) of the HE stimuli was 7.5 (*SD* = 1.4) and of the LE stimuli was 3.6 (*SD* = 1.7). Examples of HE snacks were crisps, cookies, cakes and candies. Examples of LE snacks were grapes, apples, bananas and mixed snack salads.

### fMRI single food choice task

**Figure 1:**
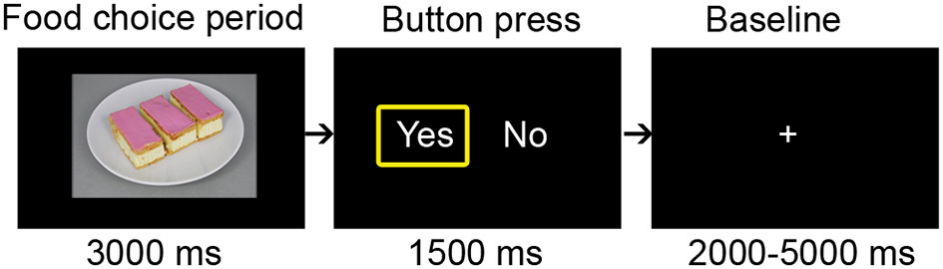
**Single food choice task trial structure**.

During the functional MRI scan, participants performed a food choice task (Figure 1). In this ask, participants made 100 choices. In every trial, they viewed one of the study stimuli (3000 ms, choice period) and subsequently had to indicate with a button press (1500 ms, button press period) whether they wanted to eat a portion of the snack or not. During the button press period the words “yes” and “no” were shown left/right (randomized) on the screen. After indicating their choice, a yellow box appeared around the yes or no. Participants were instructed to make their choice already during the period that the image was shown. To ensure that their choices were actually made in direct response to the food pictures, the button press period was so short that it only allowed them to locate whether they had to push the left or right button. The choice trials were interspersed with a random interval (2000 and 5000 ms). At the beginning, halfway (after 50 trials) and at the end an additional baseline period of 30,000 ms was included in the task.

In order to make the choices realistic, participants were instructed that one of the trials counted for real and that they would receive a portion of the snack chosen in that trial at the end of the study session. Eating a HE snack after a short period of fasting is not congruent with the participants' goal to watch their weight. Therefore, self-regulatory success was defined as the percentage of rejected HE snacks.

### Behavioral data analysis

In the food choice task trials were nested within participants. Therefore, a series of two-level logistic regression analyses were performed to investigate how tastiness and stimulus category (HE or LE) related to choice (chosen or not chosen). The statistical program R (packages lme4 and languageR) was used to perform multi-level regression analyses.

### fMRI data

#### Image acquisition and preprocessing

MRI scanning was performed on a 3 Tesla scanner (Philips Achieva, Philips Healthcare, Best, The Netherlands), equipped with an 8-channel SENSE head coil. A T_1_-weighted structural image was acquired at a resolution of 1 × 1 × 1 mm (*TR* = 8.4 ms, *TE* = 3.8 ms, total scan duration = 284 s). Functional scans were acquired with a 2D-EPI sequence (TR/TE = 1400/23 ms, flip angle = 70°, nr slices = 30, voxel size = 4 × 4 × 4 mm). The total number of volumes (540–580) acquired differed between participants because of the random inter-trial interval.

Data were preprocessed and analyzed using the SPM8 software package (Wellcome Department of Imaging Neuroscience, London, United Kingdom) ran with MATLAB R2012A (The Mathworks Inc, Natick, MA). Functional images were realigned to the first image of the time series. Functional and structural images were co-registered and normalized (retaining 4 × 4 × 4 mm voxels) to MNI space (Evans et al., 1993) by using linear and nonlinear transformations. The data were smoothed with an isotropic Gaussian kernel of 8 mm full width at half maximum.

#### Participant level analyses

Statistical maps were generated for each participant by fitting a boxcar function to the time series, convolved with the canonical hemodynamic response function. Data were high-pass filtered with a cutoff of 128 s.

Two models were fitted. Four conditions were modeled in the first model: the HE choice periods, the LE choice periods, the button press screen, and the practice trial and missed trials. To establish brain regions that respond differently to HE and LE choice periods, we performed a mean subtraction analysis between HE and LE choice periods, resulting in a contrast image of HE minus LE choice periods and a contrast image of LE minus HE choice periods. Furthermore, a contrast image of HE choice periods vs. baseline and a contrast image of LE choice periods vs. baseline were calculated.

The second model was constructed to identify brain regions in which activation correlates with tastiness. The same four conditions as in the first model were modeled. A parametric regressor with the self-reported tastiness ratings of the respective snacks was added to the HE and LE choice periods. To identify in which brain regions activation correlates with tastiness, the following two contrast images were calculated: to establish the brain regions that were related with tastiness ratings during HE choice periods we conducted a parametric modulation analysis with the tastiness ratings during HE choice periods; to establish the brain regions that were related with tastiness ratings during LE choice periods we conducted a parametric modulation analysis with the tastiness ratings during LE choice periods.

#### Group level analyses

To determine which brain regions showed differential activation for HE and LE choice periods, the contrast images of HE minus LE choice periods (and vice versa) were entered into one-sample *t*-test analyses. To determine brain regions in which activation was positively or negatively related with self-reported tastiness ratings during HE and LE choice periods, the respective contrast images were entered into one-sample *t*-tests. To establish in which brain regions individual differences in self-regulatory success (i.e., the proportion of accepted or refused HE snacks) were related with the neural activation during the choice, the contrast images of HE choice periods vs. baseline were entered into two one-sample *t*-tests with as covariate the proportion of accepted and refused HE snacks, respectively. Also, the contrast images of LE choice periods versus baseline were entered into two one-sample *t*-tests with as covariate the proportion of accepted and refused LE snacks, respectively.

To be as objective as possible in our selection of Regions of interest (ROIs), we took them from a meta-analysis on the neural response to food cues that included more than 20 studies (van der Laan et al., 2011): posterior fusiform gyrus, inferior frontal gyrus—orbital part, insular cortex, superior parietal gyrus, middle occipital gyrus, amygdala, calcarine sulcus, lingual gyrus, inferior parietal gyrus, parahippocampal gyrus, (hypo)thalamus, ventral striatum, culmen, middle frontal gyrus, and inferior temporal gyrus. ROI masks were generated using the AAL-atlas (Tzourio-Mazoyer et al., 2002) as implemented in the WFU-pickatlas toolbox (Maldjian et al., 2003). For ROIs a statistical threshold of *p* < 0.05 Family Wise Error (FWE) corrected over the ROI volume (i.e., small volume correction) was used. For completeness, we employed no additional extent threshold for ROIs. For additional statistical rigor, a Bonferroni correction should be done for the number of ROIs (15), resulting in a statistical threshold of *p* < 0.003 FWE-corrected over the ROI volume. However, considering the criticism of Bonferroni being too conservative with large numbers of tests and similar expected effects across tests (non-independence) (see e.g., Perneger, 1998), we also report results that did not survive this additional Bonferroni correction. For regions other than those of a-priori interest, we report clusters significant at a stricter statistical threshold of p < 0.001 uncorrected and a cluster extent *k* > 12, in line with other studies in the field (e.g., Martin et al., 2010; Demos et al., 2012; Seo et al., 2013; Stice et al., 2013; Van den Bosch et al., 2014).

## Results

### Behavioral results

**Figure 2:**
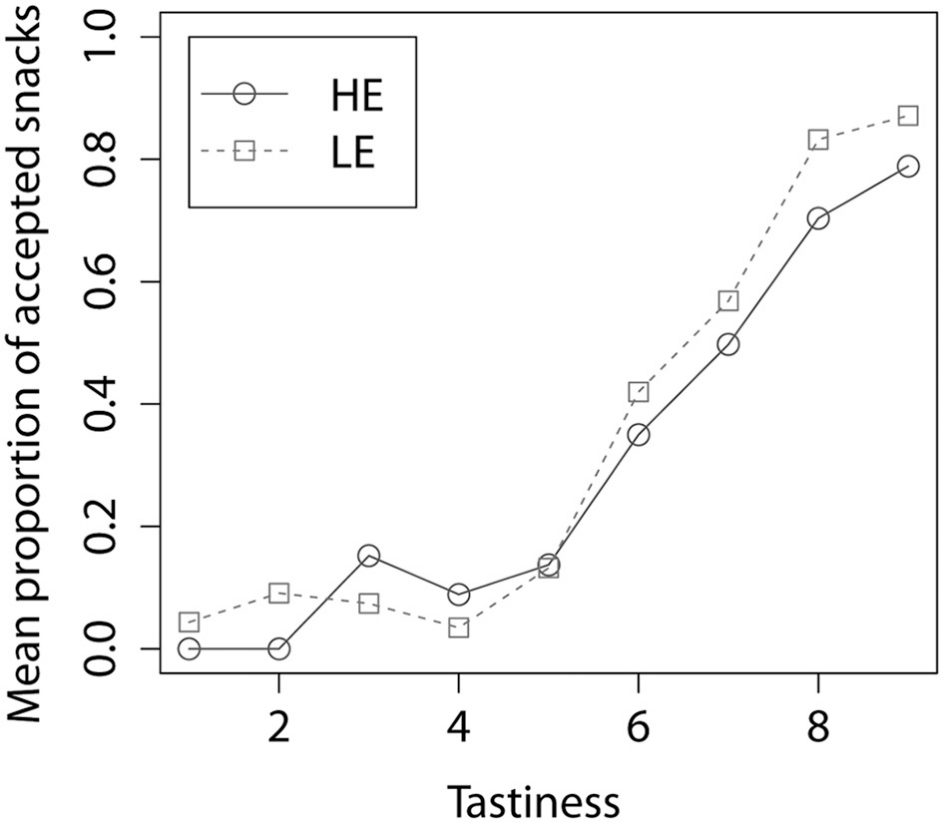
**Proportion of accepted snacks by tastiness rating, for HE and LE snacks**.

A two-level logistic regression model (Table 1) with as outcome choice (1 = accept, 0 = refuse) and as predictors energy content (LE or HE), and self-reported tastiness of the stimulus, revealed that LE snacks were accepted significantly more often (mean % ± *SD*: 62 ± 17) than HE (48 ± 24), and the more tasty a snack was (regardless of energy content) the higher the likelihood that it would be accepted. Figure 2 shows the proportion of accepted snacks per tastiness rating, for HE and LE snacks.

**Table 1:** **Multi-level logistic regression results: energy content and self-reported tastiness predict choice**.

**Model effect**	**Estimate**	***SE***	***t*-value**	***P***
**Fixed effects**				
Intercept	−6.95	0.43	−16.1	<0.001
Tastiness rating	1.00	0.05	20.0	<0.001
Energy content (HE/LE)	0.51	0.12	4.4	<0.001
**Random effects**	**Variance**	***SD***		
Intercept (level 2 subject)	1.11	1.05		
**Log-likelihood model**	−932			
**AIC**	1871			

Tastiness, rated on a 9-point scale ranging from 1 = very untasty to 9 = very tasty, did not differ significantly between LE (mean ± *SD*: 7.0 ± 0.7) and HE (6.7 ± 0.9) food categories (*p* = 0.19).

### fMRI results^1^

#### HE vs. LE choice periods

**Figure 3:**
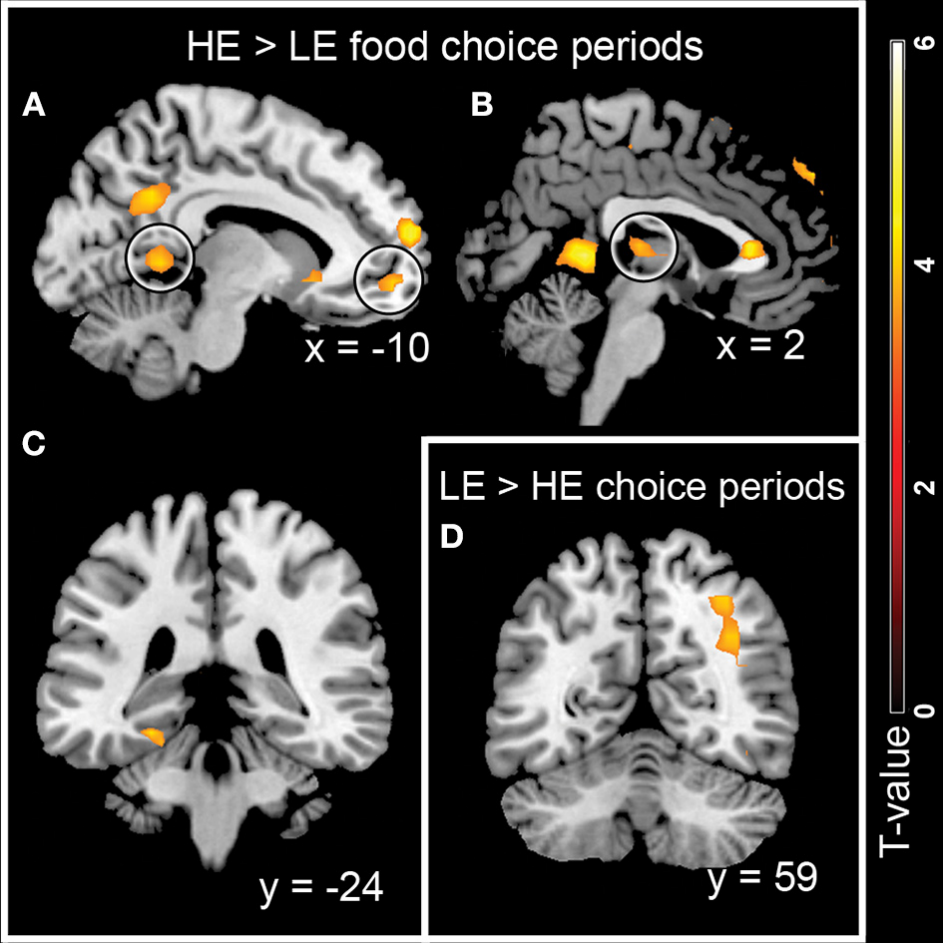
**Brain regions activated during HE vs. LE food choice periods. (A–C)** Regions stronger activated during HE food choices: clusters in **(A)** calcarine sulcus and orbital part of superior frontal gyrus, **(B)** caudate and **(C)** parahippocampal gyrus. **(D)** Brain region stronger activated during LE food choice periods: cluster in superior parietal gyrus.

Several brain regions, including the orbital part of the right superior frontal gyrus (medial to middle OFC), the left lingual gyrus, the left parahippocampal gyrus, the left calcarine sulcus, and the left caudate (marginally significant), were activated stronger in response to HE compared to LE food choices (Table 2, Figures 3A–C). Clusters in the right superior parietal gyrus were activated more strongly during LE compared to HE choice periods (Table 2, Figure 3D).

**Table 2:** **Brain regions differentially activated in response to HE and LE choice periods**.

**Brain region**	**Side^a^**	***x***	***y***	***z***	**Cluster size**	***Z*-value**	***p*^b^_FWE_**
**HE vs. LE CHOICE PERIODS**							
***ROIs*^b^**							
Calcarine sulcus	L	−6	−48	6	3	3.43	0.033
Caudate	L	−6	−16	−6	4	2.96	0.058
Superior frontal gyrus, orbital part	R	18	56	−2	7	3.18	0.018
Lingual gyrus	L	−6	−48	2	4	3.34	0.042
Parahippocampal gyrus	L	−26	−36	−14	3	3.08	0.047
***Whole brain*^c^**							
Cerebellum/lingual gyrus	R/L	−2	−44	6	24	3.56	N.A.
**LE vs. HE CHOICE PERIODS**							
***ROIs*^b^**							
Superior parietal gyrus	R	34	−56	54	1	2.99	0.031
Superior parietal gyrus	R	30	−64	50	2	2.84	0.049

a L, left hemisphere; R, right hemisphere.

b Peaks reported are significant at p < 0.05 FWE-corrected on ROI level.

c Peaks of clusters significant at p < 0.001 uncorrected, k > 12 voxels are reported.

#### Parametric modulation by tastiness

There were no brain regions in which activation was positively modulated by tastiness during HE choice periods. Activation in the orbital part (lateral OFC) and a more superior part of the middle frontal gyrus, the opercular part of the inferior frontal gyrus, and the precuneus was negatively modulated by tastiness in HE choice periods (Table 3). There were no brain regions in which activation during LE choice periods was positively or negatively modulated by tastiness.

**Table 3:** **Brain regions of which activation during HE food choice periods was negatively related to tastiness (parametric modulation)**.

**Brain region**	**Side^a^**	***x***	***y***	***z***	**Cluster size**	***Z*-value**	***p*^b^_FWE_**
**ROIs^b^**							
Middle frontal gyrus, orbital part	R	34	48	−2	8	3.13	0.048
Middle frontal gyrus	R	46	32	34	15	3.81	0.028
Inferior frontal gyrus, opercular part	R	30	4	34	2	3.82	0.009
**WHOLE BRAIN^c^**							
Precuneus	L	−6	−64	46	34	4.03	N.A.
	L	−18	−52	46		3.97	N.A.

a L, left hemisphere; R, right hemisphere.

b Peaks reported are significant at p < 0.05 FWE-corrected on ROI level.

a Peaks of clusters significant at p < 0.001 uncorrected, k > 12 voxels are reported.

#### Brain regions in which activation covaries with self-regulatory success

**Figure 4:**
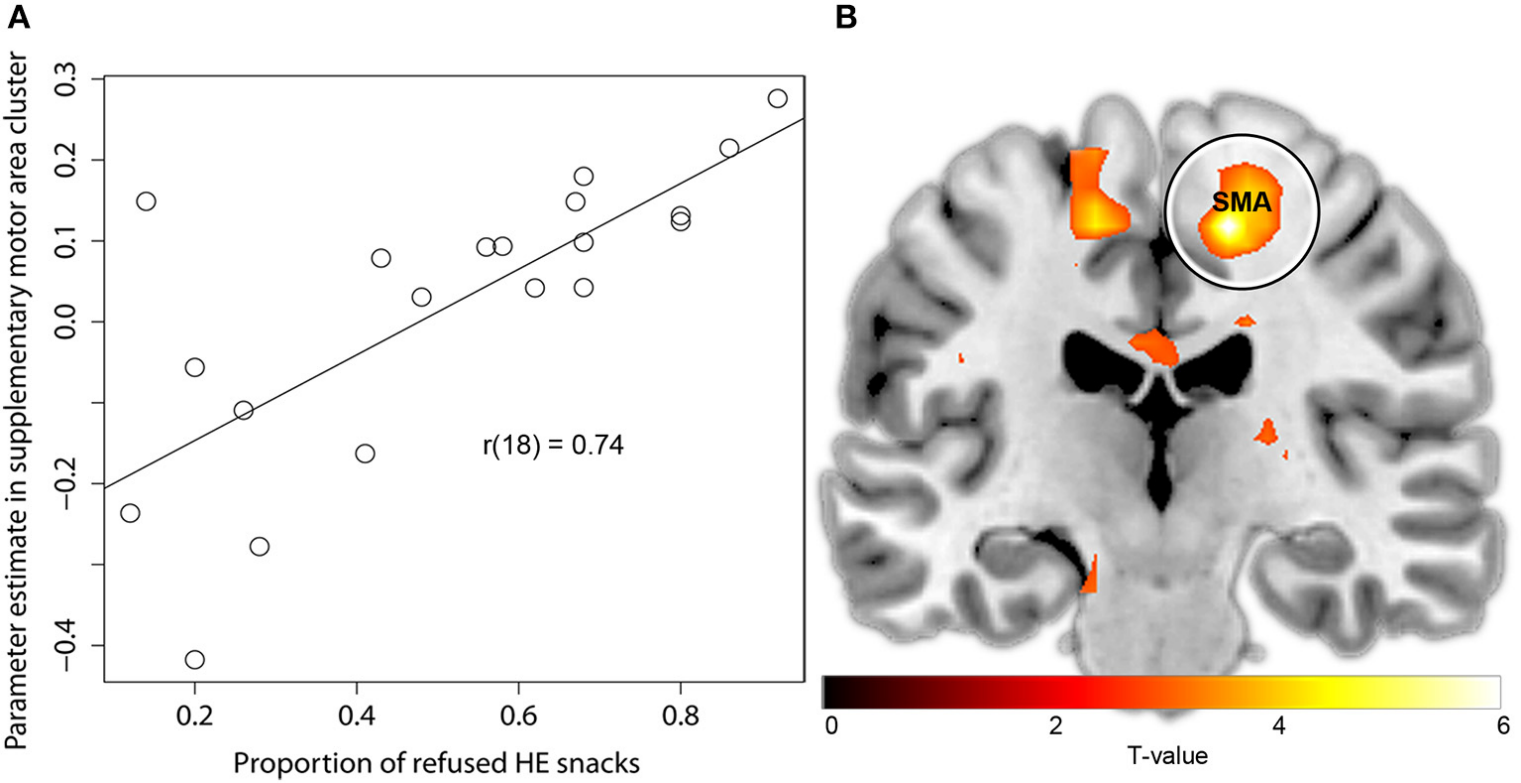
**Activation in the right SMA during the HE choice periods covaries with self-regulatory success. (A)** Plot showing correlation between parameter estimate HE vs. baseline in right SMA cluster and the proportion of rejected HE snacks. **(B)** Brain regions in which activation covaries with proportion of rejected HE snacks. Circle indicates right SMA cluster. For visualization purposes, fMRI-results are thresholded at *T* > 2.87.

There were no brain regions in which activation during HE choice periods covaried positively with the proportion of accepted HE snacks. Activation in the angular gyrus, the supplementary motor area (SMA), the middle occipital gyrus, and the cerebellum during the HE choice periods covaried significantly (positive) with the proportion of rejected HE snacks (Table 4, Figure 4). Thus, participants who rejected more HE snacks had significantly stronger activation in these areas during HE choice periods.

**Table 4:** **Brain regions of which activation during HE food choice periods covaries positively with the proportion of rejected HE snacks**.

**Brain region**	**Side^a^**	***x***	***y***	***z***	**Cluster size**	***Z*-value**
**WHOLE BRAIN^b^^,^^c^**						
Angular gyrus	R	34	−48	26	25	5.25
Supplementary motor area	R	14	−20	54	20	4.66
Middle occipital gyrus	L	−42	−68	6	13	3.98
Cerebellum	R	26	−76	−38	18	3.47
	R	34	−72	−34		3.47
	R	42	−58	−34		3.00

a L, left hemisphere; R, right hemisphere.

b There were no peaks significant in the ROI analysis (p < 0.05 FWE-corrected on ROI level).

c Peaks of clusters significant at p < 0.001, k > 12 voxels are reported.

To rule out the alternative explanation that participants who rejected more HE snacks had a lower preference for HE snacks and that activation in the identified regions reflected this, we repeated the analysis while controlling for individual differences in preference for HE snacks. To this end, the participants' mean tastiness rating of HE snacks was added as first and the proportion of rejected HE snacks as second covariate. The clusters in the SMA [*Z* = 3.94, MNI (14, −20, 54)] and cerebellum [*Z* = 4.03, MNI (−30, −80, −34)] still significantly covaried with the proportion of rejected HE snacks, indicating that activation in these regions was not due to a lower general preference for HE snacks, but rather due to self-regulatory success.

There were no brain regions of which activation during LE choice periods significantly covaried with the proportion of accepted or rejected LE snacks.

## Discussion

Our study aims were to investigate in weight-concerned women which brain regions are activated during HE and LE food choices, and to assess in which brain regions activation varied with tastiness and individual differences in self-regulatory success. On average, participants were at best moderately successful in choosing in line with their long-term goal since they accepted almost 50% of the HE snacks. The behavioral results revealed that both tastiness and energy content (independently) influenced the likelihood that a snack was accepted or refused. The finding that weight-concerned women refused more HE than LE snacks, or equivalently, accepted more LE than HE snacks which were equal in tastiness, underlines that decision-making for HE and LE snacks constitutes more than only tastiness considerations. While the number of accepted HE snacks is indicative of the extent to which someone adheres to her weight-watching goal, the number of accepted LE is not: eating an LE snack does not contribute to limiting energy intake when this snack does not replace a HE snack that would otherwise be eaten.

The finding that HE snacks were refused more often than equally liked LE snacks might suggest that the participants employed self-control to resist the HE snacks. In line with self-regulation theory, exposure to HE snacks might have supported the need to employ self-control to inhibit the initial tendency to accept them (Fishbach et al., 2003). Another explanation for why more LE than HE snacks were accepted could be that participants perceived LE snacks as healthy and therefore eating them conduces health. Other studies have shown that people eat more of foods which are assumed to be healthy (Wansink and Chandon, 2006; Provencher et al., 2009) and another study even showed that people erroneously believe that eating healthy foods in addition to unhealthy ones can decrease total calorie count (Chernev, 2011).

It is important to note that the HE and LE snacks did not differ significantly in average tastiness in our study. This is a vital advantage of our study compared to many earlier studies in which brain responses to energy content and tastiness were confounded due to the higher tastiness of the HE foods (van der Laan et al., 2011) Yet, although equally tasty, we found that HE food choices still elicited stronger activation in a cluster in the medial/middle OFC that has consistently been shown to activate during processing and evaluating rewarding stimuli (Elliott et al., 2000; Kringelbach, 2005; Hampshire et al., 2012). Furthermore, a cluster in the caudate tended (borderline significant) to activate stronger during HE food choice. This region plays a prominent role in reward processing as it responds to both valence and saliency of rewards (Carlezon and Thomas, 2009; Litt et al., 2011). Finally, a cluster in the parahippocampal gyrus activated more strongly during HE (vs. LE) food choice. Parahippocampal gyrus activation has been shown to predict subsequent consumer choice and is thought to reflect the expected reward value of a stimulus (Tusche et al., 2010). This region has also been shown to activate during choices in which an immediate (and not when a delayed) reward was available (McClure et al., 2004). Since participants were slightly hungry due to the 2 h fast before the scan, it could be that HE snacks had a higher momentary biological reward value due to the energy content, which translates into activation in the OFC, the caudate and the parahippocampal gyrus. This is also in line with the finding that hunger modulates the neural activation to visual food cues in the parahippocampal gyrus (van der Laan et al., 2011). To our knowledge, we are the first to investigate the differential neural response during food choices concerning HE and LE snacks in weight-concerned women. Altogether, our findings suggest that for weight-concerned women, HE snacks might have a higher reward than LE snacks, even when they are equally tasty.

Since it is particularly difficult to resist a HE snack when it is very tasty, it was expected that choice-related activation in brain regions involved in conflict and self-control would vary with tastiness. Therefore, our second aim was to investigate in which brain regions activation was parametrically modulated by tastiness. In line with the behavioral finding that highly tasty HE snacks were indeed resisted less often, we found that activation in the opercular part of the inferior frontal gyrus and the orbital part of the middle frontal gyrus (lateral OFC) varied negatively with tastiness during HE choice. That is, activation in these areas was lower for tastier HE snacks. The opercular part of the inferior frontal gyrus has previously been shown to activate during response inhibition (Aron et al., 2014). Studies have shown that the lateral OFC (in contrast to the medial OFC which activates in response to rewards) is involved in response inhibition (Elliott et al., 2000; Kringelbach, 2005). Thus, lower activation in these regions might be explained by their failure to inhibit desire for highly tasty HE snacks. An explanation for why we only identified this cluster for the HE and not for LE snacks might be that LE snacks do not pose a threat to the long-term weight watching goal, and therefore do not elicit inhibitory responses (neither when high, nor when low in tastiness).

Our third aim was to investigate whether the neural response during food choices covaried with self-regulatory success as indicated by the number of refused HE snacks. We found that participants who rejected more HE snacks showed stronger activation during HE food choices in several brain regions, including the SMA. The SMA receives inputs from the striatum, through the pre-SMA, and projects to the primary motor cortex, leading to action (Haggard, 2008, 2009). Although the (pre-)SMA has repeatedly been shown to activate during inhibitory processes relating to food and monetary stimuli (Hendrick et al., 2012; Hollmann et al., 2012; Ma et al., 2012; Pawliczek et al., 2013), the exact function of the SMA in response inhibition is relatively poor understood. Classically, it was thought that the SMA merely serves as an intermediate between higher cognitive areas and the motor cortex, which would imply that SMA activation is just reflective of decisions made elsewhere (e.g., in the OFC, Nachev, 2006). Accordingly, it could be argued that SMA functioning is crucial for self-control since functioning in this brain region determines whether the choices made in higher cognitive areas can actually be executed. On the other hand, the pre-SMA and SMA are increasingly being implicated in immediate executive control (Oliveri et al., 2003; Nachev, 2006; Hollmann et al., 2012). In line with this notion, it has been found that stimulating the SMA with transcranial magnetic stimulation (TMS) appeared to influence excitability of the motor cortex differently during exposure to emotionally unpleasant compared to neutral visual cues (Oliveri et al., 2003). This would suggest that the SMA is not merely an intermediate, but that rather that transmission from the SMA to the motor cortex depends on the emotional valence of the cue that triggers the behavior. In our study, the stronger SMA activation during food choice in participants that refused more HE snacks, may reflect inhibition of their initial tendency to accept HE snacks (as indicated by the stronger reward-related striatal and OFC response to HE compared to LE snacks in this study). Future research using techniques (like TMS) that (temporarily) disrupt the function of the SMA and higher cognitive areas should elucidate the causal role of the SMA in food-related self-control.

From theory it follows that having a long-term goal is a prerequisite for perceiving an internal conflict in response to a self-control dilemma (e.g., Fishbach et al., 2003). For this reason, we included participants which were weight-concerned according to self-reports. We cannot claim, however, that results are specific for this group. Therefore, it is of high interest to repeat this paradigm in a non-weight-concerned population. By comparing our results with a group of non-weight-concerned women, we could rule out whether the effects seen in the present study are general effects that occur also in non-weight-concerned women or whether they are specific for weight-concerned women in which the self-control dilemma is relevant. Our study population consisted of women with a normal weight and therefore their motivation for weight-concern might not arise from medical or health reasons. Rather, since earlier studies showed a clear link between worries about appearance and weight-concerns/restraint (Putterman and Linden, 2004, 2006; O'Brien et al., 2007; de Ridder et al., 2014) we think that the high level of self-reported weight-concerns in our study population might indicate an intention to lose weight for cosmetic reasons or general concerns about healthy eating. Although our population was of normal weight, research has shown that normal-weight individuals who report high weight-concerns might be at increased risk for gaining weight (French et al., 1994; Mann et al., 2007). Therefore, normal weight females reporting high levels of weight-concerns are a very important population to focus on in research and weight-maintenance interventions.

A limitation of our study is that we did not control for possible effects of menstrual cycle phase. This may have introduced some variation in brain responses. However, since 16 of the 20 participants used hormonal contraceptives, which reduce hormonal fluctuations, we do not think that this has significantly biased our findings.

To conclude, our findings indicate that HE snacks constitute a higher reward than LE snacks, for weight-concerned women, even when they are equally tasty. This might explain why it is so hard to resist HE snacks. The negative association between brain activation in inhibitory areas and tastiness suggests that inhibition fails when HE snacks are very tasty. Finally, women who better adhere to their long term weight-watching goal show increased SMA activation during food choices concerning HE snacks, which emphasizes the need for future research assessing whether SMA functioning plays a role in the control of food intake.

## Author contributions

Laura Nynke van der Laan, Paul A. M. Smeets, and Denise T. D. de Ridder conceived and designed the experiment. Laura Nynke van der Laan collected and analyzed the data. Laura Nynke van der Laan, Paul A. M. Smeets, and Denise T. D. de Ridder interpreted the data. Laura Nynke van der Laan wrote the manuscript and Paul A. M. Smeets and Denise T. D. de Ridder provided critical revisions. Max A. Viergever contributed to the final version of the manuscript by interpreting results, reviewing and critically revising text. All authors approved the final version for submission and agreed to be accountable to for all aspects of the work.

### Conflict of interest statement

The authors declare that the research was conducted in the absence of any commercial or financial relationships that could be construed as a potential conflict of interest.

## Footnotes

^1^ None of the clusters survived the additional Bonferroni correction. Results reported for ROI are significant at *p* < 0.05 FWE corrected over the ROI volume.
